# The Association of Sociodemographic Factors, Postictal Symptoms, and Medical History With Seizure Type in Patients With Epilepsy: A Cross-Sectional Study

**DOI:** 10.7759/cureus.39763

**Published:** 2023-05-31

**Authors:** Reem Alyoubi, Summayah A Kobeisy, Mazen Basheikh, Rayan A Al-Sharief, Majed M Al-Hayani, Yousof O Rayes, Atheer Alharthi, Anas S Alyazidi, Nuha Alrayes, Haythum O Tayeb

**Affiliations:** 1 Department of Pediatrics, King Abdulaziz University Hospital, Jeddah, SAU; 2 Department of Pediatrics, Dr. Soliman Fakeeh Hospital, Jeddah, SAU; 3 Department of Internal Medicine, University of Jeddah, Jeddah, SAU; 4 Department of Medicine, King Abdulaziz University, Rabigh, SAU; 5 Department of Medicine and Surgery, Umm Al-Qura University, Makkah, SAU; 6 Department of Medicine, King Abdulaziz University Hospital, Jeddah, SAU; 7 Department of Medicine, King Abdulaziz University, Jeddah, SAU; 8 Department of Medical Laboratory Sciences, Faculty of Applied Medical Sciences, King Abdulaziz University, Jeddah, SAU; 9 Department of Medicine, Neuroscience Research Unit, Faculty of Medicine, King Abdulaziz University, Jeddah, SAU; 10 Department of Neurology, Faculty of Medicine, King Abdulaziz University, Jeddah, SAU

**Keywords:** sociodemographic, epidemiology, seizure, saudi arabia, epilepsy

## Abstract

Background

Approximately 50 million people globally suffer from epilepsy. The prevalence of epilepsy in Saudi Arabia has been reported at 6.5 per 1,000 persons, affecting nearly 1% of the entire population. However, limited data is available in the country regarding the sociodemographic factors affecting epilepsy and its associated postictal symptoms, which may lead to stigmatization and negatively impact patients.

Methods

A cross-sectional study was conducted at King Abdulaziz University Hospital (KAUH) in a survey format. Ethical approval was obtained from the Research Ethics Committee of the Faculty of Medicine at King Abdulaziz University. The study population included patients with epilepsy who visited King Abdulaziz University Hospital’s outpatient neurology clinics from October 2021 to March 2022.

Results

The study participants’ average age at the time of the first seizure was 16.5 years, with patients experiencing seizures as early as within the first year of life and as late as 70 years of age. Patients who had had their first seizure during the first year of life did not have any schooling (p<0.0001) and had learning difficulties (p<0.00001). Focal onset impaired awareness seizures were significantly associated with motor weakness (p=0.023) and mood alterations (p=0.014), while postictal fear, anxiety or panic, and sleep disruption were statistically significant for focal onset aware seizures (p=0.015 and p=0.050).

Conclusion

This study highlights the sociodemographic differences between patients in Saudi Arabia and in other areas. It may also point to novel findings regarding the postictal symptoms associated with the various seizure types.

## Introduction

Epilepsy is a chronic brain disease causing transient abnormal cerebral activity that may lead to seizures, unusual behavior, sensations, or loss of consciousness. It is estimated that epilepsy affects 50 million people worldwide with an annual cumulative incidence of 67.77 per 100,000 persons [1-3]. Its prevalence is expected to increase due to increased life expectancy and advancements in the medical field that lead to improved survival rates of those with traumatic brain injuries, perinatal injury, central nervous system infections, and stroke, which are all causes of epilepsy [1]. The disease burden of epilepsy differs across countries and accounts for roughly 1% of the disease burden worldwide [2]. Almost 80% of people with epilepsy come from low- to middle-income countries; mortality rates due to the disease in these countries are also higher [2,4]. In 2017, the International League Against Epilepsy (ILAE) reclassified seizure nomenclature to better aid the clinician in describing the disorder and help patients in understanding their condition [3]. Seizures previously referred to as simple partial and complex partial are now known as focal onset aware and focal onset impaired awareness seizures [4]. Epilepsy is prevalent in Saudi Arabia, with an incidence rate of 6.5 per 1,000 people [5]. Generally in the Middle East and specifically in Saudi Arabia, limited data is available regarding sociodemographic factors affecting epilepsy and public awareness levels regarding the disease, which may lead to stigmatization and negatively impact those affected [6-9]. The aim of this study is to explore sociodemographic characterizations of epilepsy patients in Saudi Arabia and report their postictal symptoms and past medical history.

## Materials and methods

Study design and ethical considerations

A cross-sectional study was conducted at King Abdulaziz University Hospital (KAUH) in a survey format distributed in the clinic and online using patients’ phone numbers in the hospital record. Ethical approval was obtained from the Research Ethics Committee of the Faculty of Medicine at King Abdulaziz University in Jeddah, Saudi Arabia (reference number: 199-22), and the guidelines outlined in the Declaration of Helsinki were followed. Furthermore, the Strengthening the Reporting of Observational Studies in Epidemiology (STROBE) checklist [10] was adopted for the present study.

Study population

The study population included patients who visited the KAUH outpatient neurology clinics from October 2021 to March 2022. Criteria for inclusion were patients previously diagnosed with epilepsy and following up in the KAUH outpatient neurology clinics during the study period. Written informed consent was required as well as parental consent for those less than 18 years old.

Sampling methodology

The survey comprised three sections. The first included a consent question, in which participants gave consent to proceed to the following section. The second included demographic data such as age, gender, and marital status. The third solicited information on seizure type, age at the time of first seizure, seizure frequency, and presence of convulsions. The occurrence of status epilepticus was also included. The survey encompassed postictal symptoms and past medical history as well as family history. Data were collected and entered into a database.

Data analysis

The data used to conduct this survey and support the findings are available upon request from the corresponding author. Statistical analysis was carried out using Statistical Package for the Social Sciences (SPSS) version 20 (IBM SPSS Statistics, Armonk, NY, USA). The level of significance (p-value) was taken at <0.05. The Bonferroni correction was used in post hoc testing of the data.

## Results

**Figure 1:**
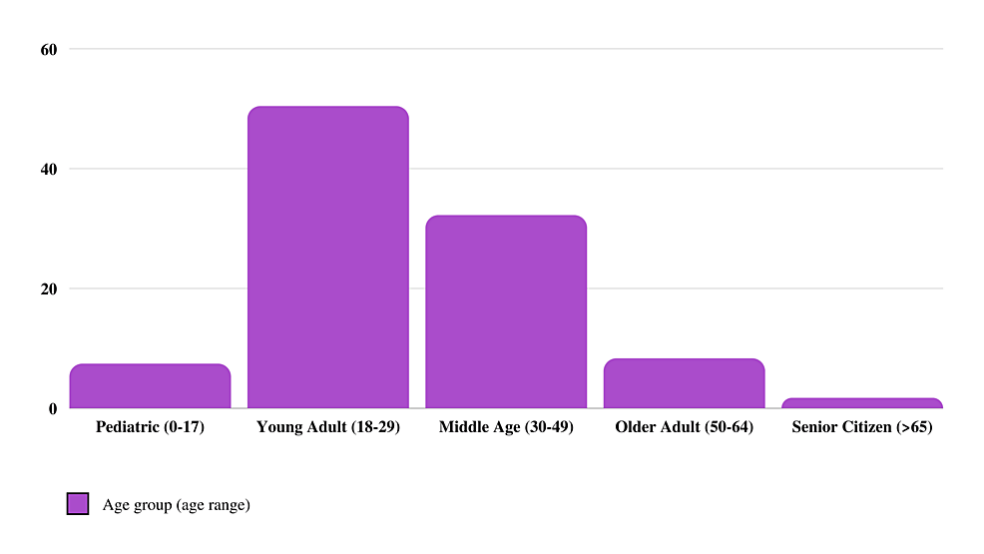
Age Group Distribution of the Study Participants Displaying a Majority of Participation by Young Adults

**Figure 2:**
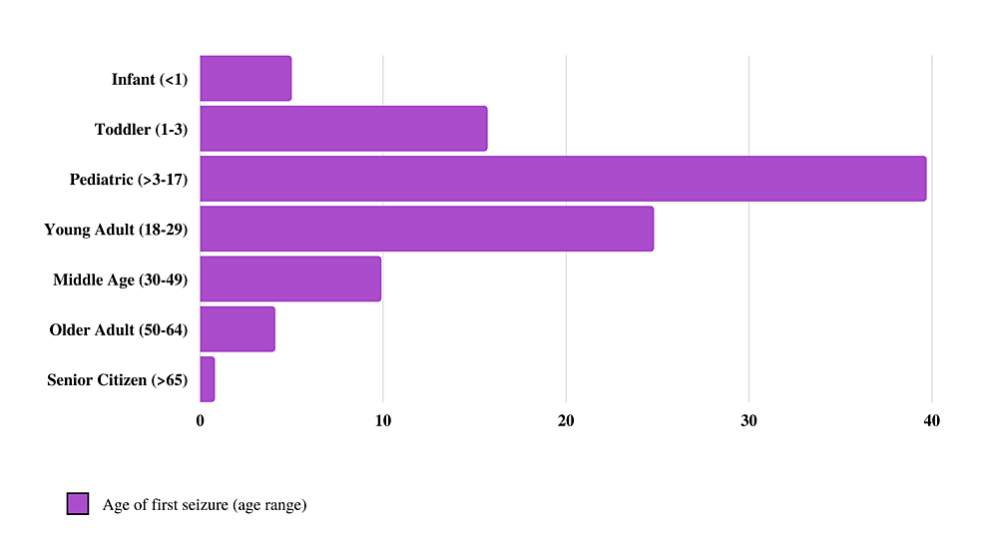
Different Age Groups According to the Reporting Age of First Seizures

There were a total of 121 study participants. Their average age was 30.5 years, with the youngest being 12 and the oldest being 76. Most patients were between the ages of 18 and 29 years (50.4%), and only two patients were in the senior citizen age group (Figure 1). The patients’ average age at the time of the first seizure was 16.5 years, with patients experiencing seizures as early as within the first year of life and as late as 70 years of age. The majority of patients had experienced their first seizure between the ages of three and 17 years (39.7%), followed by those between the ages of 18 and 29 years (24.8%). The majority of the cohort (60.4%) had experienced their first seizure below 18 years of age (Figure 2). Females constituted the majority of the sample (52.9%), and the majority were nonsmokers (81.8%). Only 34.8% had received higher education, and 62% were single. Regarding seizure type, the most common was generalized onset (73.6%), followed by focal onset impaired awareness (52.9%) and focal onset awareness (34.7%). The incidence of generalized onset seizures among those with focal onset impaired awareness was higher than those with focal onset awareness seizures (79.7% and 69%, respectively). Of the group, 29.8% reported having experienced status epilepticus, and 19% experienced daily seizures (Table 1).

**Table 1:** Demographic Characteristics of Patients SD: standard deviation

Demographic characteristics	
Age group	Number (%)
Pediatric (0-17)	9 (7.4)
Young adult (18-29)	61 (50.4)
Middle age (30-49)	39 (32.2)
Older adult (50-64)	10 (8.3)
Senior citizen (>65)	2 (1.7)
Age	
Mean±SD	30.5±12.6
Minimum-maximum	12-76
Age at first seizure	
Mean±SD	16.5±14.5
Minimum-maximum	0-70
Age at first seizure	Number (%)
Infant (<1)	6 (5)
Toddler (1-3)	19 (15.7)
Pediatric (>3-17)	48 (39.7)
Young adult (18-29)	30 (24.8)
Middle age (30-49)	12 (9.9)
Older adult (50-64)	5 (4.1)
Senior citizen (>65)	1 (0.8)
Gender	Number (%)
Male	57 (47.1)
Female	64 (52.9)
Smoking status	Number (%)
Smoker	22 (18.2)
Nonsmoker	99 (81.8)
Educational status	Number (%)
No education	18 (14.9)
Less than high school diploma	18 (14.9)
High school diploma	43 (35.5)
Bachelor’s degree	36 (29.8)
Postgraduate degree	6 (5)
Marital status	Number (%)
Single	75 (62)
Married	45 (37.2)
Divorced	1 (0.8)
Focal onset aware seizure	Number (%)
Yes	42 (34.7)
No	79 (65.3)
Focal onset impaired awareness seizure	Number (%)
Yes	64 (52.9)
No	57 (47.1)
Generalized seizure	Number (%)
Yes	89 (73.6)
No	32 (26.4)
Status epilepticus	Number (%)
Yes	36 (29.8)
No	85 (70.2)
Seizure frequency	Number (%)
Never	7 (5.8)
Daily	23 (19)
Weekly	14 (11.6)
Monthly	39 (28.9)
Annually	38 (31.4)

Of the postictal symptoms, motor weakness was the most common (66.1%), followed by confusion (62.8%) and sleep disruption (52.9%). Only 9% of the patients experienced postictal hallucinations (Table 2).

**Table 2:** Detailed Postictal Symptom Data

Postictal symptoms	
Confusion	Number (%)
Yes	76 (62.8)
No	45 (37.2)
Motor weakness	Number (%)
Yes	80 (66.1)
No	41 (33.9)
Sensory loss	Number (%)
Yes	35 (28.9)
No	86 (71.1)
Disruptive behavior	Number (%)
Yes	27 (22.3)
No	94 (77.7)
Hallucination	Number (%)
Yes	11 (9.1)
No	110 (90.9)
Fear, anxiety, or panic	Number (%)
Yes	23 (19)
No	98 (81)
Mood alterations	Number (%)
Yes	36 (29.8)
No	85 (70.2)
Sleep disruption	Number (%)
Yes	64 (52.9)
No	57 (47.1)

Nearly one-fourth of patients reported a family history of epilepsy (24.8%), and 36.4% had a family history of consanguinity. However, learning difficulties and developmental delay were reported at 13.2% and 11.6%, respectively, in each of the above groups. A little more than a third of patients were documented to have previous head trauma (33.9%), while only 5.8% had had a central nervous system infection, and 12.4% had experienced a febrile convulsion (Table 3).

**Table 3:** Detailed Medical History of Patients

Past medical history	
Neurological disorder	Number (%)
Yes	30 (24.8)
No	91 (75.2)
Birth difficulty	Number (%)
Yes	12 (9.9)
No	109 (90.1)
Developmental delay	Number (%)
Yes	14 (11.6)
No	107 (88.4)
Learning difficulties	Number (%)
Yes	16 (13.2)
No	105 (86.8)
Head trauma	Number (%)
Yes	41 (33.9)
No	80 (66.1)
Febrile convulsion	Number (%)
Yes	15 (12.4)
No	106 (87.6)
Meningitis/encephalitis	Number (%)
Yes	7 (5.8)
No	114 (94.2)
Family history of epilepsy	Number (%)
Yes	30 (24.8)
No	91 (75.2)
Family history of consanguinity	Number (%)
Yes	44 (36.4)
No	77 (63.6)
Psychiatric comorbidity	Number (%)
Yes	9 (7.4)
No	112 (92.6)
Psychiatric follow-up	Number (%)
Yes	13 (10.7)
No	108 (89.3)
Psychiatric treatment	Number (%)
Yes	5 (4.1)
No	116 (95.9)

Age of first seizure was statistically significant for marital status, patient’s educational status, history of neurological disorder, history of learning difficulties, history of head trauma, history of febrile convulsion, and patient’s smoking status. Using the Bonferroni adjusted significance value, only those who had had their first seizure during the first year of life were statistically found to have no educational background (p<0.0001) and learning difficulties (p<0.00001).

## Discussion

Worldwide, epilepsy is known to have a bimodal age distribution, peaking in infancy and adults above the age of 50 [11]. However, 39.7% of patients in this study experienced their first seizure between the ages of >3 and 17 years. Only 5% were less than one year of age, and 4.9% were 50 years and above at the time of their first seizure. Unexpectedly, 60.4% of the participants had experienced their first seizure before the age of 18. A little more than half of those studied were female (52.9%). This is contrary to results reported in the literature where the incidence of epilepsy is higher in males than in females [12]. The great majority (62.8%) of this study’s cohort were either single or divorced. Divorce rates in women with epilepsy are higher than those of men, and people with epilepsy are more likely to face marital stigma and divorce [13]. Only 18.2% of those studied were found to be smokers, compared to a study in the United States that demonstrated that 24% of epilepsy patients were smokers [14]. These differences may highlight regional distinctions in the sociodemographic backgrounds of those affected with epilepsy. Focal onset impaired awareness seizures were more common among study participants than focal onset aware seizures (52.9% and 34.7%, respectively). However, the majority reported generalized onset seizures (73.6%). Among epilepsy patients, focal seizures are the most common, with focal onset impaired awareness seizures being the most common subtype [15]. Of those surveyed, 29.8% reported having status epilepticus. A systematic review found that 30%-44% of those with status epilepticus have a history of epilepsy, thus denoting a strong relationship between those with epilepsy and status epilepticus [16]. Regarding seizure frequency, almost one-fifth reported daily seizures. This may indicate poor compliance with medication, treatment-refractory seizure, or inadequate medical management.

The ill-defined period after a seizure, otherwise known as the postictal state, is not well understood; there is limited research regarding this topic [17,18]. Motor weakness (66.1%), confusion (62.8%), and sleep disruption (52.9%) were the most common postictal symptoms found. Among those studied, there were significant statistical differences between types of seizure and the associated postictal symptoms. Altered consciousness, headache, paresis, and psychiatric symptoms are common postictal symptoms associated with generalized onset seizures [18]. Headaches and migraines after seizures are common in both adults and pediatrics but more so among the latter [16]. Participants with generalized onset seizures in this study were found to have statistically significant postictal confusion (p=0.001), motor weakness (p≤0.0001), and sensory loss (p=0.017). Focal onset impaired awareness seizures were associated with motor weakness (p=0.023) and mood alterations (p=0.014), while postictal fear, anxiety, or panic, and sleep disruption were statistically significant for focal onset aware seizures (p=0.015 and p=0.050, respectively). Studies have reported postictal unresponsiveness as the most common symptom with an occurrence of 96%. Those with refractory focal epilepsy are most likely to have postictal memory impairment (66%) [18]. Interestingly, the literature contains sparse data comparing postictal symptoms of focal awareness and focal impaired awareness seizures. To the best of the author’s knowledge, this may be a groundbreaking finding regarding the differences between these two seizure types.

Literature has shown that patients with epilepsy are more likely to have developmental delays and learning setbacks, especially in school, than those without the disease [19-22]. Those studied and reported to have generalized onset seizures were found to have a statistically significant history of developmental delay and learning difficulties (p=0.017 and p=0.010, respectively). Patients with focal onset aware seizures were also found to have a statistically important history of learning difficulties (p=0.045), while those with focal onset impaired awareness seizures had a history of febrile convulsions and head trauma (p=0.025 and p=0.010, respectively). Other studies have also shown a correlation between febrile seizures and epilepsy [23]. Surprisingly, 10%-20% of epilepsy cases are due to post-traumatic brain injury; however, little is known about the neuronal damage progression, and no treatment prophylaxis exists to prevent late-onset seizures from occurring after head trauma [24]. Patients who had their first seizure during the first year of life did not have any schooling (p<0.0001) and had learning difficulties (p<0.00001). This is similar to other studies demonstrating that people with epilepsy experience educational obstacles and scholastic difficulties in catching up to their peers [19]. Those who had their first seizure during the toddler years were associated with febrile convulsions (p=0.0004), while those who experienced their first seizure between the ages of 50 and 64 were found to have a history of neurological disorders (p=0.0001). Surprisingly, a family history of epilepsy and consanguinity were not significantly related to any seizure subtype in this study, although nearly one-fourth of patients were reported to have a family history of epilepsy (24.8%) and 36.4% had one of consanguinity. Several studies have reported family history and consanguinity as strong factors for epilepsy [25,26]. Perhaps the location of this study, an urban and modernized region of Saudi Arabia where many people are educated, poses a limitation regarding the true significance of family history, consanguinity, and epilepsy. It is common for patients with epilepsy to suffer psychiatric comorbidities. Therefore, it is prudent that medical management of epilepsy, as well as psychiatric therapy and follow-up, be provided to patients that need these services [27]. Only 7.4% of patients included in this study reported having a psychiatric disorder, and only 4.1% were on treatment. This low proportion may be due to underreporting or stigmatization of mental disorders, psychiatric treatment, and epilepsy [28].

## Conclusions

This study highlights the sociodemographic differences between patients in Saudi Arabia and in other areas. It may also point to novel findings regarding the postictal symptoms associated with the various seizure subtypes. Further investigations are needed to support these findings. Other similar studies may be conducted in rural areas of the country to examine the association of family history and consanguinity with epilepsy. Additional awareness needs to be raised regarding the benefits of mental health, and psychiatric support should be readily provided to those with epilepsy.
